# Porcine Respirovirus 1 Suppresses Host Type I Interferon Production and the JAK-STAT Signaling Pathway

**DOI:** 10.3390/v15051176

**Published:** 2023-05-16

**Authors:** Yanhua Li, Chenxi Li

**Affiliations:** 1 College of Veterinary Medicine, Yangzhou University, Yangzhou 225009, China; 2 Department of Diagnostic Medicine & Pathobiology, Kansas State University, 1800 Denison Avenue, Manhattan, KS 66506, USA; 3 Comparative Medicine Research Institute, Yangzhou University, Yangzhou 225009, China; 4 Jiangsu Co-Innovation Center for Prevention and Control of Important Animal Infectious Diseases and Zoonoses, Yangzhou 225009, China

**Keywords:** PRV1, PPIV1, V protein, type I IFN, IFN signaling pathway, RIG-I ubiquitination

## Abstract

Porcine respirovirus 1 (PRV1), first reported in Hong Kong, is currently widely spread in several countries. Our knowledge of the clinical significance and the pathogenicity of this virus is still limited. In this study, we studied the interactions between PRV1 and host innate immune responses. PRV1 exhibited strong inhibitory effects on the production of interferon (IFN), ISG15, and RIG-I induced by SeV infection. Our data generated in vitro suggest that multiple viral proteins can suppress host type I interferon production and signaling, including N, M, and P/C/V/W. The P gene products disrupt both IRF3 and NF-κB dependent type I IFN production and block type I IFN signaling pathway by sequestering STAT1 in the cytoplasm. The V protein disrupts both MDA5 signaling and RIG-I signaling through interaction with TRIM25 and RIG-I, V protein blocks RIG-I polyubiquitination, which is required for RIG-I activation. V protein also binds to MDA5, which may contribute to its inhibitory effect on MDA5 signaling. These findings indicate that PRV1 antagonizes host innate immune responses using various mechanisms, which provides important insights into the pathogenicity of PRV1.

## 1. Introduction

Paramyxoviruses that belong to the *Paramyxoviridae* family within the *Mononegavirales* order [1] are negative-sense single-stranded RNA viruses. They can infect humans and a range of animals, including livestock species such as cattle, pigs, and poultry. According to current ICTV taxonomy, the *orthoparamyxovirinae* subfamily in the *Paramyxoviridae* family can be classified into eight genera: *Respirovirus*, *Rubulavirus*, *Avularvirus*, *Morbillivirus*, *Aquaparamyxovirus*, *Ferlavirus*, *Henipavirus*, and *Salemvirus*. The genus *Respirovirus* consists of seven recognized species: *Bovine respirovirus 3* (previously called bovine parainfluenza virus 3; BPIV3), *Caprine respirovirus 3*, *Human respirovirus* (previously called human parainfluenza virus 1; HPIV1), *Human respirovirus 3* (previously called human parainfluenza virus 3; HPIV3), *Murine respirovirus* (previously called Sendai Virus; SeV), *Porcine respirovirus 1* (previously called porcine parainfluenza virus 1; PRV1) and *Squirrel respirovirus* [1,2]. Recently, *Porcine parainfluenza virus 2* was proposed to be a new species in the *Respirovirus* genus by a Chinese research group based on bioinformatic analysis of their obtained viral genomes [3]. Paramyxoviruses cause variable clinical symptoms in terms of severity and presentation. Many of these paramyxoviruses are zoonotic and lead to fatal diseases in humans, such as the Nipah virus and Hendra virus [4,5]. Swine are the primary reservoir of porcine rubulavirus and PRV1 and are intermediate hosts for the Nipah virus [6,7].

PRV1 was initially identified in nasopharyngeal samples of slaughterhouse pigs in Hong Kong, though virus isolation failed [8]. Based on molecular epidemiological and serological analyses, PRV1 has been detected in American, European, and Asian countries [3,9,10,11,12,13,14,15], suggesting the global distribution of this emerging virus. PRV1 is usually associated with clinical signs in naturally infected pigs, such as coughing, minor sneezing, and serous nasal discharge [9], and the co-infection with the influenza virus and porcine reproductive and respiratory syndrome virus in a respiratory disease may enhance its replication [12]. Virus isolation from pig nasal swab samples collected in the U.S. herd has been reported [16,17]. In an experimental pig challenge study [18], the replication of PRV1 was detected in the upper and lower respiratory tracts of four-week-old conventional nursery pigs and six-week-old cesarean-derived/colostrum-deprived pigs, which caused mild lung lesions in conventional pigs. No significant clinical respiratory disease was observed with the single infection of PRV1 in those pigs. To date, the role of PRV1 infection in the pig respiratory disease complex is largely unknown.

As a member of the Respirovirus genus, PRV1 contains a single-stranded negative-sense RNA genome of approximately 15 kb, which encodes for at least six proteins in the conserved order 3′-N-P-M-F-HN-L-5′ [1,8]. N, P, M, F, HN, and L represent the nucleocapsid, phosphoprotein, matrix protein, fusion protein, hemagglutinin-neuraminidase proteins, and polymerase protein, respectively. Both F and HN proteins are involved in receptor binding, possess neutralizing epitopes, and are the most genetically diverse viral proteins. In addition, the P gene encodes three more accessory proteins, C and V/W proteins, through leaking scanning and mRNA editing, respectively [8,17]. Based on the previous studies in paramyxoviruses [19], those accessory proteins may be critical for the antagonism of host innate immune responses during PRV1 infection.

Type I interferon is a key aspect of the host’s innate immune response, serving as the first defense line against viral infection. IFNα/β exhibits antiviral, antiproliferative, and immunomodulatory functions [20]. The pathogen-associated molecular patterns of RNA viruses are mainly recognized by cytosolic pattern-recognition receptors (PRRs), including retinoic acid-inducible protein I (RIG-I) and melanoma differentiation-associated gene 5 (MDA5). For instance, activated RIG-I caspase recruitment domains are associated with IFNβ promoter stimulator 1 (IPS-1) to activate the downstream kinases TBK1 and IKKε, which leads to the phosphorylation and activation of transcription factors IRF3 and NF-κB. With the coordinated activation of these transcription factors, a transcriptionally competent enhanceosome is assembled to induce type I IFN production [21]. The secreted type I IFN binds their receptors on cell surfaces, which results in the activation of the JAK-STAT signaling pathway [22,23]. Janus-activated kinases (JAKs) activated by the receptor-ligand interaction lead to the phosphorylation of signal transducers and activators of transcription (STATs). The heterotrimeric complex interferon-stimulated gene factor 3 (ISGF3) is assembled by the phosphorylated STATs (STAT1 and STAT2) association with IRF9 in the cytoplasm. ISGF3 further translocates to the nucleus, where it binds to IFN-stimulated response elements (ISRE) in the promoter and induces the transcription of IFN-stimulated genes (ISGs). These ISGs function as antiviral effectors to combat viral replication and further spread [24]. To bypass host restriction and establish infection, paramyxoviruses employ many strategies to evade type I interferon (IFN) production and signaling pathways, in which the accessory proteins play critical roles [19]. However, whether PRV1 evades type I IFN production, signaling pathways, and potential mechanisms are unknown.

In this study, we studied the interactions between PRV1 and host type I IFN pathways. In viral-infected LLC-MK2 cells, PRV1 exhibited strong inhibitory effects on the production of IFNβ and ISGs. The mechanisms utilized by KS 17-258 strain to evade host type I interferon production and signaling pathway were further investigated. Several viral proteins are involved in suppressing type I IFN production and signaling pathways, including N, M, and P/C/V/W. The P gene products disrupt both IRF3 and NF-κB-dependent type I IFN production and block type I IFN signaling by sequestering STAT1 in the cytoplasm. Through interaction with TRIM25 and RIG-I, V protein blocks RIG-I polyubiquitination, which is required for RIG-I activation. V protein also interacts with MDA5, which may contribute to its inhibitory effect on MDA5 signaling.

## 2. Materials and Methods

### 2.1. Cells, Viruses, and Reagents

LLC-MK2 cells (ATCC^®^ CCL-7.1™) purchased from ATCC (Manassas, VA, USA) were used for virus infection experiments. HEK-293T cells were used for ectopic expression experiments. The cells were cultured with minimum essential medium (MEM) (Invitrogen, Waltham, MA, USA) supplemented with 10% heat-inactivated fetal bovine serum (Sigma-Aldrich, St. Louis, MO, USA) and antibiotics (100 units/mL of penicillin, 100 µg/mL of streptomycin, and 0.25 µg/mL of fungizone) at 37 °C with 5% CO_2_. The Sendai virus (SeV) Cantell strain, grown in embryonated chicken eggs, was used to stimulate the type I IFN response in the cell culture system. A PRV1 virus (GenBank accession No. MH396493) isolated previously [17] was passaged in LLC-MK2 cells, and the culture supernatant was harvested as virus stock for subsequent experiments.

The following antibodies were used in this study, including monoclonal antibody (mAb) 75-3 against PRV1 F protein, anti-RIG-I mAb (Alme-1) (AdipoGen life sciences, San Diego, CA, USA), anti-ISG15 mAb (F-9) (Santa Cruz Biotechnology, Dallas, TX, USA), anti-STAT1α mAb (C-111) (Santa Cruz Biotechnology, Dallas, TX, USA), anti-pSTAT1 mAb (pY701.4A) (Santa Cruz Biotechnology, Dallas, TX, USA), SV40 T Ag antibody (Pab 101) (Santa Cruz Biotechnology, Dallas, TX, USA), anti-GST mAb (B14) (Santa Cruz Biotechnology, Dallas, TX, USA), anti-FLAG tag mAb M2 (Sigma-Aldrich, St. Louis, MO, USA), anti-GAPDH mAb clone GAPDH-71.1 (Sigma-Aldrich, St. Louis, MO, USA), and anti-c-Myc mAb 9E10 (DSHB, Iowa City, IA, USA).

A panel of plasmids was generated for the ectopic expression of eight PRV1 proteins (N-P/C/V/W-M-F-HN). The coding sequence of the P gene was amplified by RT-PCR and inserted into a eukaryotic expression vector p3xFLAG-Myc-CMV-24 and designated as p3xFLAG-P. To create plasmids expressing V and W proteins, one or two G insertion at the mRNA editing site (AAAAAGGG) of p3xFLAG-P was introduced by QuickChange^TM^ site-directed mutagenesis kit (Agilent Technologies, Santa Clara, CA, USA). Plasmids for the expression of N, M, F, and HN were generated by cloning the corresponding coding sequences in the pCAGGS vector, and a FLAG-tag was fused at the C-terminus of these proteins. All primers for RT-PCR amplification of viral genes are listed in Table 1. The p125-Luc and p55-CIB-Luc reporter plasmids kindly provided by Takashi Fujita [25] were used to monitor IFNβ promoter activation, and the pNF-κB-Luc reporter plasmid from Agilent Technologies was used to study NF-κB dependent pathway. The pISRE-Luc reporter plasmid from Agilent Technologies was employed to evaluate the activation of the type I IFN signaling pathway. The pRL-SV40 plasmid expressing a Renilla luciferase under the control of a simian virus 40 (SV40) promoter (Promega, Madison, WI, USA) was utilized to normalize transfection efficiency. The pEFneo-RIG-I, pEFneo-MDA5, and pEFneo-IKKε were kindly provided by Dr. Bin Gotoh [26]. The pCAGGS-HA-MDA5 was constructed by cloning HA-tagged MDA5 into the pCAGGS vector. The pCAGGS-myc-TRIM25 was constructed by cloning Myc-tagged human TRIM25 into the pCAGGS vector. The pCAGGS-GST was constructed by cloning the glutathione S-transferase (GST) gene into the pCAGGS vector. The pCAGGS-GST-RIG-IN, which expresses the 2CARD domain of human RIG-I as GST-fusion protein, was constructed by cloning the GST-RIG-IN fusion gene into the pCAGGS vector. The pcDNA3.1(+)-HA-Ub was provided by Domenico Tortorella (Mount Sinai School of Medicine, New York, NY, USA) [27].

### 2.2. Immunofluorescence Assay (IFA)

The nucleus translocation of STAT1 was investigated by immunofluorescence assay. Briefly, HEK-293T cells were co-transfected with pEGFP-STAT1 and a plasmid expressing PRV1 P protein or another accessory protein (C/V/W). At 24 hpt, cells were treated with IFNα for 2 h or mock-treated. Cell monolayers fixed with 4% paraformaldehyde were incubated with primary antibody at an appropriate dilution at 37 °C for 1 h and then extensively washed with 1x PBS several times to remove the unbound antibodies. The Alexa Fluor^®^ 488 conjugated goat anti-mouse IgG(H + L) was used as a secondary antibody at an appropriate dilution. After 45 min of incubation at 37 °C, cell monolayers were washed with 1× PBS 5 times and stained with 4′,6-diamidino-2-phenylindole (DAPI) (Invitrogen, Carlsbad, CA, USA). Confocal microscopy was performed using a Zeiss LSM880 confocal laser-scanning microscope (Oberkochen, Germany).

### 2.3. Quantitative RT-PCR

To evaluate type I IFN production, a quantitative RT-PCR was performed to assess the expression of IFNβ at the mRNA level. Briefly, total RNA was extracted from cell lysate in TRIzol reagent (ThermoFisher Scientific, Waltham, MA, USA) according to the manufacturer’s instructions. DNase treatment with TURBO DNA-free™ Kit (ThermoFisher Scientific, Waltham, MA, USA) was performed to remove genomic DNA contamination. A total of 1 μg RNA was used to prepare a one-step RT-PCR reaction which was formulated with 5 µL TaqMan™ Fast Virus 1-Step Master Mix (ThermoFisher Scientific, Waltham, MA, USA) and 1 μL of each predesigned primers/probe set (ThermoFisher Scientific, Waltham, MA, USA) for monkey IFNβ and GAPDH. Reactions were completed on the CFX96 Real-Time PCR system (Bio-Rad) under the following conditions: 5 min of 50 °C for reverse transcription, 20 s of 95 for RT inactivation/initial denaturation, 40 amplification cycles of 30 s of 95 °C, and 30 s of 60 °C. The mRNA expression levels of IFNβ were normalized to the endogenous GAPDH mRNA level.

### 2.4. Dual-Luciferase Reporter Assay

For luciferase reporter assay, HEK-293T cells were transfected with 0.5 µg plasmid DNA of p125-luc, p55-CIB-luc, pNF-κB-luc or pISRE-luc, 20 ng plasmid DNA of pRL-SV40, and 0.5 µg plasmid DNA expressing PRV1 proteins, or empty vector (EV). TransIT^®^-LT1 Transfection Reagent (Mirus Bio LLC, Madison, WI, USA) was utilized for all transfection experiments following the manufacturer’s instructions. At 24 h post-transfection, cells were stimulated by infection with SeV at 100 HA unit per ml or IFNβ at 100 IU/mL for 16 h. Cell lysates were harvested and subjected to a reporter assay using a dual-luciferase reporter system (Promega, Madison, WI, USA) according to the manufacturer’s instructions. Firefly or Renilla luciferase activity was measured in FLUOstar Omega (BMG Labtech, Ortenberg, Germany). The relative luminescence unit (RLU) was calculated by normalizing the firefly luciferase to Renilla luciferase activities. The relative luciferase activity of EV with stimulation was set as 100%. Western blot analysis was conducted to evaluate viral protein expression. In addition, the cytotoxicity of viral proteins was evaluated using Cell Counting Kit-8 (Dojindo molecular technologies, Inc., Rockville, MD, USA) following the manufacturer’s instructions.

### 2.5. Immunoprecipitation Assay (IP)

An immunoprecipitation assay was performed to evaluate the interactions between PRV1 V protein and cellular factors, including MDA5, RIG-I, and TRIM25. Briefly, HEK-293T cells were co-transfected with a plasmid expressing V protein with FLAG-tag and a plasmid expressing MDA5, RIG-I, or TRIM25, and an empty vector was included as a control. At 36 hpt, cells were harvested with Pierce IP Lysis Buffer (Thermo Fisher Scientific, Waltham, MA, USA) supplemented with a protease inhibitor cocktail (Sigma-Aldrich, St. Louis, MO, USA). Cell lysates were clarified by centrifugation at 15,000 g at 4 °C for 15 min and used for IP with anti-FLAG mAb M2. IP was performed using the Pierce™ Classic Magnetic IP/Co-IP Kit (Thermo Fisher Scientific, Waltham, MA, USA) according to the manufacturer’s instructions. Cellular factors contained in the immunoprecipitated protein complex were detected by Western blot analysis using mAbs.

### 2.6. GST Pull-Down Assay

The effect of V protein on the ubiquitination of RIG-I was studied by a GST pull-down assay. Briefly, HEK-293T cells were transfected with the following plasmid combinations, including pCAGGS-GST/pCAGGS/pcDNA-HA-Ub, pCAGGS-GST-RIG-IN/pCAGGS/pcDNA-HA-Ub, and pCAGGS-GST-RIG-IN/pCAGGS-Flag-V/pcDNA-HA-Ub. At 24 hpi, cells were harvested with Pierce IP Lysis Buffer (Thermo Fisher Scientific, Waltham, MA, USA) supplemented with a protease inhibitor cocktail (Sigma-Aldrich, St. Louis, MO, USA). Cell lysates were clarified by centrifugation at 15,000 g at 4 °C for 15 min and used for GST pull-down with the Pierce™ GST Protein Interaction Pull-Down Kit (Thermo Fisher Scientific, Waltham, MA, USA) according to the manufacturer’s instructions. The ubiquitination status of GST-RIG-IN and the V protein in the pull-down protein complex were determined by Western blot analysis using a mAb against HA-tag and a mAb against FLAG-tag, respectively.

### 2.7. Western Blot Analysis

To evaluate protein expression in cells, Western blot analysis was performed as described previously [28]. Briefly, cells were harvested with passive lysis buffer of Dual-Luciferase^®^ Reporter Assay System (Promega, Madison, WI, USA) or Pierce IP Lysis Buffer (Thermo Fisher Scientific, Waltham, MA, USA) supplemented with protease inhibitor cocktail (Sigma-Aldrich, St. Louis, MO, USA). Cell lysates clarified by centrifugation at 15,000 g for 15 min were mixed with Laemmli sample buffer (4×) and heated at 95 °C for 6 min. After being separated in denatured Tris-glycine gel using sodium dodecyl sulfate-polyacrylamide gel electrophoresis (SDS-PAGE), all proteins were transferred onto a nitrocellulose membrane using wet electro-transfer. The membrane was blocked with 5% skim milk at 4 °C overnight. The primary antibody at an appropriate dilution was added and incubated at room temperature for 1 h. After three washes with 1× PBS containing 0.05% TWEEN 20 (PBST), the membrane was incubated with the secondary antibody, IRDye^®^ 800CW Goat anti-Mouse IgG (H + L) or/and IRDye^®^ 680RD goat anti-Rabbit IgG (H + L) (LI-COR Biosciences, Lincoln, NE, USA) at an appropriate dilution for 1 h at room temperature. The membrane was extensively washed with PBST to remove any unbound antibodies and then scanned with a digital image system (Odyssey infrared imaging system; LI-COR Biosciences, Lincoln, NE, USA).

### 2.8. Statistical Analysis

Statistical analyses were performed with GraphPad Prism software 8.0. Data are presented as mean values ± SD from the biological replicates. The mean values were compared using two-tailed Student’s *t*-tests or ANOVA with Tukey’s post hoc test and considered significantly different when *p*-value < 0.05.

## 3. Results

### 3.1. PRV1 Infection Antagonizes Type I IFN Production and Signaling Pathway

**Figure 1:**
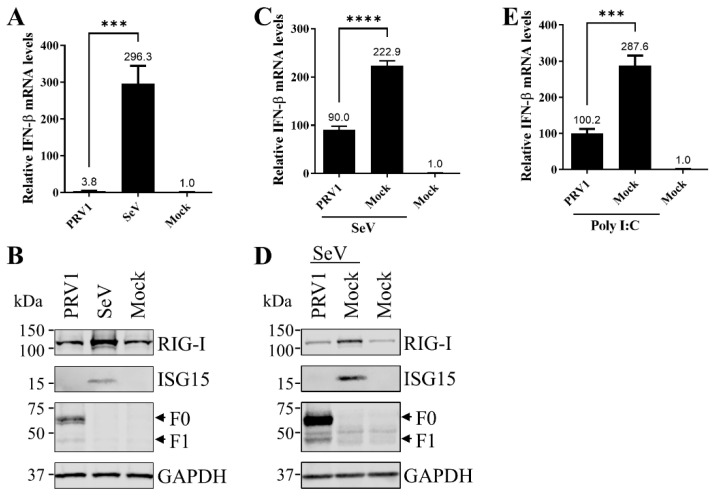
The expression IFNβ and ISGs in LLC-MK2 cells infected with PRV1 (KS 17-258 strain). (**A**,**B**) PRV1 infection was unable to stimulate IFN-β production. LLC-MK2 cells were infected with KS 17-258 at an MOI of 1. LLC-MK2 cells infected with 100 HA units of SeV and with mock infection were used as the positive and negative control. At 24 hpi, cells were harvested with TRIzol reagent or IP lysis buffer. IFNβ expression at the mRNA level was evaluated by real-time RT-PCR with total cellular RNA. The relative mRNA expression level of IFN-β was normalized to the mRNA level of GAPDH, and the relative IFN-β mRNA level of negative control was set as 1 (**A**). The expression of two ISGs (RIG-I and ISG15) was assessed by Western blot analysis with monoclonal antibodies. F protein was detected to monitor KS 17-258 infection, and housekeeping gene GAPDH was detected as the loading control (**B**). (**C**,**D**) PRV1 infection suppressed IFNβ production. LLC-MK2 cells were infected with KS 17-258 at an MOI of 1 or mock-infected. At 24 hpi, cells were infected with 100 HA units of SeV to induce type I IFN production. Naïve LLC-MK2 cells were included as the negative control. After 12 h, cells were harvested with TRIzol reagent or IP lysis buffer, and IFNβ expression at the mRNA level was evaluated by real-time RT-PCR. The relative mRNA expression level of IFN-β was normalized to the mRNA level of GAPDH, and the relative IFNβ mRNA level of negative control was set as 1 (**C**). The expression of two ISGs (RIG-I and ISG15) was assessed by Western blot analysis with monoclonal antibodies. F protein was detected to monitor KS 17-258 infection, and housekeeping gene GAPDH was detected as the loading control (**D**). (**E**) PRV1 infection suppressed IFNβ production stimulated by poly I:C transfection. LLC-MK2 cells were infected with KS 17-258 at an MOI of 1 or mock-infected. At 24 hpi, cells were transfected with poly I:C at 2 μg/mL for 8 h. Naïve LLC-MK2 cells were included as the negative control. Cells were harvested with TRIzol reagent, and IFNβ expression at the mRNA level was evaluated by real-time RT-PCR. The relative mRNA expression level of IFN-β was normalized to the mRNA level of GAPDH, and the relative IFNβ mRNA level of negative control was set as 1. ***, *p*-value < 0.001; ****, *p*-value < 0.0001.

The infection of a respirovirus is always associated with poor innate immune responses [19]. In this study, we assessed the ability of PRV1 to stimulate or suppress cellular innate immune responses by evaluating the induction of type I IFN and JAK-STAT signaling pathways. We first determined innate immune responses stimulated by PRV1 infection by evaluating the expression of IFNβ. PRV1 virus was used to infect LLC-MK2 cells at an MOI of 1.0, and the SeV Cantell strain was used as a positive control for type I IFN induction. The induction of type I IFN was evaluated by the mRNA expression level of IFNβ quantified by qRT-PCR. As shown in Figure 1A, in comparison with mock infection, PRV1 infection induces limited upregulation (3.8-fold) of IFNβ mRNA expression, while SeV infection induced 296.3-fold upregulation of IFNβ mRNA expression. The stimulation of two representative interferon-stimulated genes (ISG), ISG15 and RIG-I, was also evaluated by Western blot analysis. In comparison with those in mock cells, much higher levels of RIG-I and ISG15 were detected in cells with SeV, but no obvious upregulation was observed in cells with PRV1 infection (Figure 1B).

Next, we determined the ability of PRV1 to suppress host type I IFN production and signaling pathways. As expected, a 222.9-fold upregulation of IFNβ mRNA expression was stimulated by SeV (Figure 1C). The IFNβ mRNA level in cells with consecutive infections by PRV1 and SeV was 90-fold higher than that of mock control cells. However, in comparison with 222.9-fold upregulation of IFNβ mRNA in cells only infected with SeV, PRV1 infection significantly attenuated the production of IFNβ. Consistent with data on IFNβ production, PRV1 infection showed an antagonism function on the expression of ISG15 and RIG-I stimulated by SeV infection (Figure 1D). The suppression effect of PRV1 infection on IFNβ production was also confirmed in cells stimulated with poly I:C (Figure 1E).

### 3.2. Multiple PRV1 Proteins Antagonize Type I IFN Production

**Figure 2:**
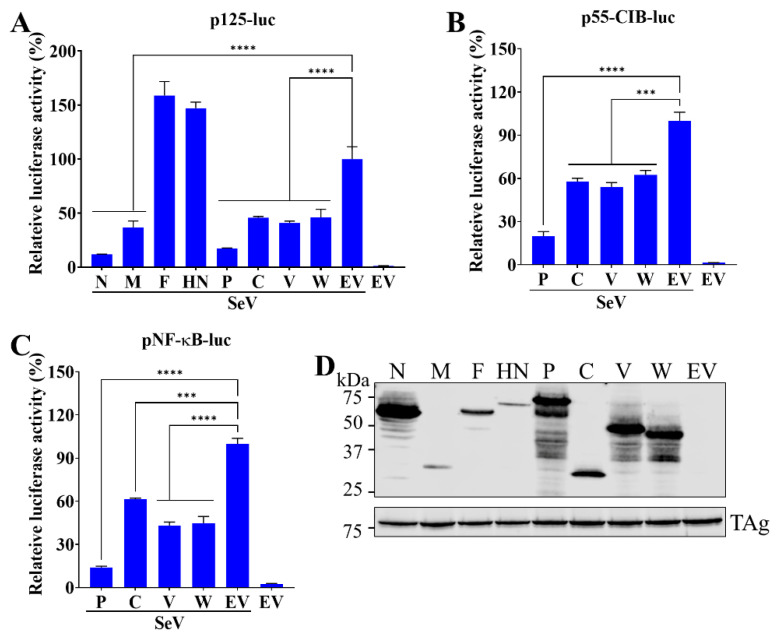
Multiple PRV1 proteins suppress IFN-β production. HEK293T cells cultured in 24-well plates were co-transfected with a plasmid expressing PRV1 proteins, or the empty vector (EV), pRL-SV40, and a luciferase reporter plasmid p125-Luc (**A**), p55-CIB-luc (**B**) or pNF-κB-luc (**C**). At 24 h post-transfection, cells were infected with 100 HA units/mL SeV for 16 h to stimulate the production of interferon. Firefly and Renilla luciferase activities were measured with cell lysate. Relative luciferase activity is defined as the ratio of firefly luciferase reporter activity to Renilla luciferase activity. The relative luciferase activity of EV with stimulation was set as 100%. Each data point shown represents a mean value from two biological replicates, and each experiment was repeated at least three times. Error bars show standard deviations of the normalized data. (**D**) The expression of PRV1 proteins in HEK-293T cells was evaluated by Western blot analysis with anti-FLAG mAb M2. The SV40 larger T antigen (TAg) was detected as the loading control. ***, *p*-value < 0.001; ****, *p*-value < 0.0001.

To identify the innate immune antagonists encoded by PRV1, all viral proteins except the L protein were cloned and ectopically expressed in HEK-293T cells. The ability of viral proteins to suppress the production of type I IFN was assessed with a reporter plasmid p125-luc, in which the firefly luciferase gene is under the control of the IFNβ promoter. As expected, the expression of firefly luciferase in cells transfected with an empty vector was stimulated by SeV infection. However, firefly luciferase expression was significantly suppressed in cells with the expression of N, M, and P gene products (Figure 2A). All four viral proteins encoded by the P gene showed a significant inhibition effect on the expression of firefly luciferase.

In the IFNβ promoter, four positive regulatory domains (PRDs) offer the binding sites for three different transcription factors: interferon regulatory factor 3 (IRF3) (PRDs I and III), nuclear factor-B (NF-κB) (PRDII), and activating protein 1 (AP1) (PRD IV). The binding of transcription factors to the promoter region and forming a so-called enhanceosome on the PRDs are required for maximal activation of the IFNβ promoter (reviewed in [29]). The signaling pathways for IFNβ dependent on IRF3 or NF-κB have been well-studied and targeted by many viral proteins to suppress host innate immune responses. To investigate which pathways can be blocked by PRV1 proteins (P, C, V, and W) encoded by the P gene, two reporter plasmids (p55-CIB-luc and pNF-κB-luc) were used. p55-CIB-luc reporter plasmid contains a firefly luciferase gene under the control of a promoter with three IRF3 binding sites, whereas pNF-κB-luc reporter plasmid contains a firefly luciferase gene under the control of an NF-κB responsive promoter with two NF-κB binding sites. SeV infection was conducted to activate type I IFN production. As expected, in HEK-293T cells without viral protein expression, SeV infection activated both IRF3 and NF-κB-dependent signaling pathways (Figure 2B,C). In contrast, the activation of both IRF3 and NF-κB-dependent pathways was effectively inhibited in HEK-293T cells with the expression of the P gene products. In addition, the expression of viral proteins was verified by Western blot analysis (Figure 2D).

### 3.3. V Protein Suppresses IFNβ Production by Targeting MDA5 and RIG-I

**Figure 3:**
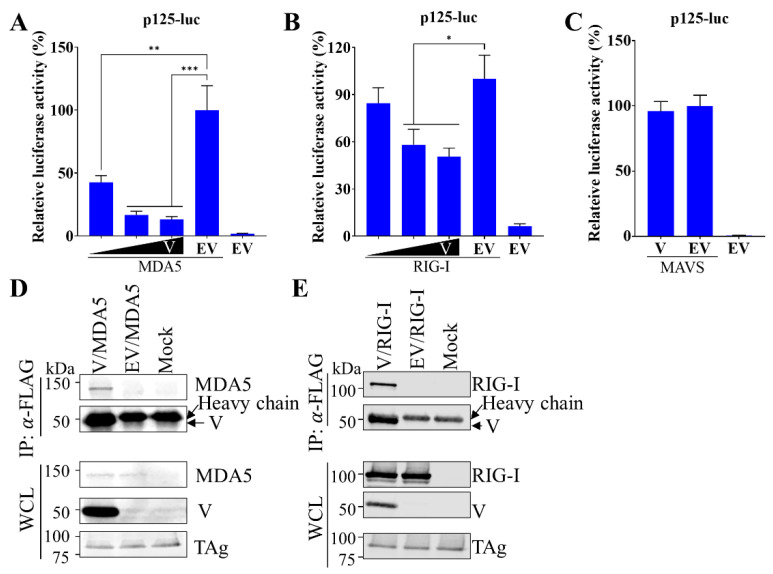
PRV1 V protein inhibits MDA5-dependent and RIG-I-dependent IFNβ production. (**A**,**B**) HEK-293T cells in 24-well plates were co-transfected with the plasmid pEFneo-MDA5 (**A**) or pEFneo-RIG-I (**B**), along with pRL-SV40, an increasing amount (0.25, 0.5, and 1.0 μg) of pCAGGS expressing V protein or the empty vector (EV), and p125-luc plasmid for 24 h. (**C**) HEK-293T cells in 24-well plates were co-transfected with the plasmid pEGFP-C3-MAVS, along with pRL-SV40, 1μg of pCAGGS expressing V protein or the empty vector (EV), and p125-luc plasmid for 24 h. Firefly and Renilla luciferase activities were measured with cell lysate. Relative luciferase activity is the ratio of firefly luciferase reporter activity to Renilla luciferase activity. The relative luciferase activity of EV with stimulation was set as 100%. Each data point shown represents a mean value from two biological replicates, and each experiment was repeated at least three times. Error bars show standard deviations of the normalized data. (**D**) The interaction between V protein and MDA5. HEK-293T cells were co-transfected with a plasmid expressing V protein or the empty vector (EV), and a plasmid expressing HA-MDA5. At 24 hpt, cell lysates were harvested for immunoprecipitation (IP) with anti-FLAG mAb M2. Western blot analysis was performed to detect V protein and MDA5 with anti-FLAG and anti-HA mAbs, respectively. (**E**) The interaction between V protein and RIG-I. HEK-293T cells were co-transfected with a plasmid expressing V protein or the empty vector (EV) and a plasmid expressing HA-RIG-I. At 24 hpt, cell lysates were harvested for immunoprecipitation (IP) with anti-FLAG mAb M2. Western blot analysis with IP eluents and whole-cell lysate (WCL) was performed to detect V protein and RIG-I with anti-FLAG and anti-HA mAbs, respectively. The SV40 larger T antigen (TAg) was detected as the loading control (**D**,**E**). *, *p*-value < 0.05; **, *p*-value < 0.01; ***, *p*-value < 0.001.

In parainfluenza viruses, the V protein functions as an antagonist to type I IFN induction by strategies targeting two RNA helicase molecules (MDA5 and RIG-I) that can directly recognize dsRNA [30,31,32,33,34,35,36,37]. Here, we hypothesized that PRV1 V protein suppresses type I IFN induction by blocking the activation of MDA5 and RIG-I. To test it, we first determined the effect of V protein on the activation of transcription from p125-luc reporter plasmid mediated by MDA5 and RIG-I. As expected, the activation of transcription was observed in cells with the overexpression of MDA5 (Figure 3A) and RIG-I (Figure 3B). In contrast, the activation by MDA5 and RIG-I was blocked by the co-expression of V protein in a dose-dependent manner. These results suggest PRV1 V protein may block IFNβ induction by targeting MDA5 and RIG-I. Another possibility is that the V protein blocked downstream steps of the MDA5 and the RIG-I-mediated signaling pathway. Since MDA5 or RIG-I is associated with MAVS caspase to activate the downstream portion of the signaling pathway, we evaluated the effect of V protein on MAVS-mediated IFNβ induction. Activated reporter gene expression in cells with co-expression of MAVS and V protein indicates that the V protein is unable to suppress MAVS-mediated IFNβ induction (Figure 3C). Taken together, the V protein appeared to suppress IFNβ induction by targeting two cytosolic receptors, MDA5 and RIG-I.

Next, we examined the interactions between V protein and MDA5/RIG-I by co-immunoprecipitation (co-IP) assays. V protein and MDA5 were co-expressed in HEK-293T cells. Using anti-FLAG mAb M2 recognizing FLAG-tagged V protein, we observed that MDA5 was coprecipitated with V protein (Figure 3D). The interaction between the V protein and RIG-I was explored similarly. As shown in Figure 3E, RIG-I was also coprecipitated with V protein. These data indicated that PRV1 V protein binds to MDA5 and RIG-I.

### 3.4. V Protein Suppresses RIG-I Ubiquitination

**Figure 4:**
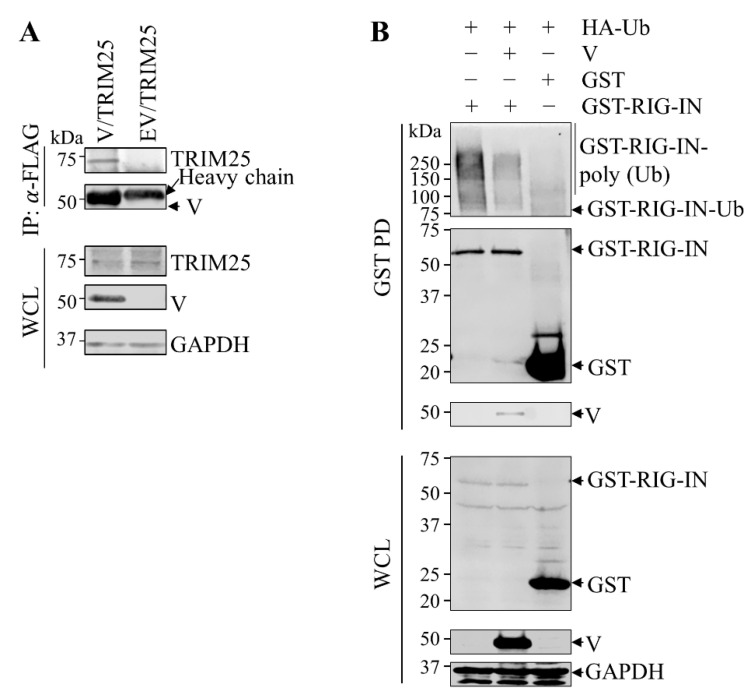
PRV1 V protein inhibits RIG-I ubiquitination. (**A**) The interaction between V protein and TRIM25. HEK-293T cells were co-transfected with a plasmid expressing V protein or the empty vector (EV), and a plasmid expressing c-MYC-TRIM25. At 24 hpt, cell lysates were harvested for immunoprecipitation (IP) with anti-FLAG mAb M2. Western blot analysis with IP eluents and whole-cell lysate (WCL) was performed to detect V protein and RIG-I with anti-FLAG and anti-c-MYC mAbs, respectively. (**B**) HEK-293T cells were transfected with plasmids expressing HA-Ub, GST, or GST-RIG-IN as indicated. Cells were also co-transfected with the empty vector or a plasmid expressing PRV1 V. A GST pulldown assay was performed. Western blot analysis was performed with anti-HA mAb to show covalent ubiquitin-modified species of GST–RIG-IN as indicated. Western blot analysis with whole-cell lysate (WCL) was performed to verify protein expression. The housekeeping gene GAPDH was detected as the loading control (**A**,**B**).

The K63-linked ubiquitination of RIG-I by the TRIM25 E3 ubiquitin ligase is required for the activation of RIG-I-mediated IFN production [38]. Here, we further explored the interaction between the V protein and TRIM25 by coexpressing these two proteins in HEK-293T cells. Co-IP was performed to analyze the interaction using anti-FLAG mAb M2 which recognizes FLAG-tagged V protein. TRIM25 was detected in the co-IP eluent by mAb against the c-MYC tag, suggesting that V protein can interact with TRIM25 (Figure 4A). Given the interaction of PRV1 V protein with TRIM25, we hypothesized that V protein interferes with the activation of RIG-I via TRIM25. To test this, the effect of V protein on RIG-I ubiquitination was evaluated by GST pulldown assay (GST-PD). The RIG-I protein consists of three distinct domains: a tandem caspase activation and recruitment domain (2CARD), a helicase domain, and a repressor domain (RD). The N-terminal RIG-I-2CARD (RIG-IN) is the functional domain that activates type I IFN production without dsRNA stimulation, whereas RD can bind 2CARD to inhibit its function [39]. The pCAGGS-GST-RIG-IN plasmid expressing a fusion protein (GST-RIG-IN) was used for GST-PD. HEK-293T cells were co-transfected plasmids expressing GST or GST-RIG-IN with the absence or the presence of PRV1 V protein, as indicated. Ubiquitin (HA-Ub) was also overexpressed in HEK-293T cells. GST-PD was performed with lysates of HEK-293T cells, and the eluent was used for Western blot analysis. GST-RIG-IN and GST proteins were detected in the eluent by an anti-GST mAb. A pattern of smear bands was detected by an anti-HA mAb in cells with only GST-RIG-IN expression. These proteins are covalently modified isoforms of GST-RIG-IN with HA-Ub. When PRV1 V was coexpressed, this ubiquitination pattern of RIG-IN was significantly reduced (Figure 4B), indicating that PRV1 V protein inhibits RIG-I ubiquitination. In addition, PRV1 V protein was also detected in GST-PD eluent, confirming that PRV1 V protein interacts with the N-terminal 2CARD domain of RIG-I. Taken together, these results indicate that PRV1 V protein suppresses type I IFN production at the step of RIG-I ubiquitination.

### 3.5. PRV1 P Gene Products Inhibit Interferon-Stimulated Gene Expression from ISRE Promoter by Blocking the Nucleus Translocation of STAT1

**Figure 5:**
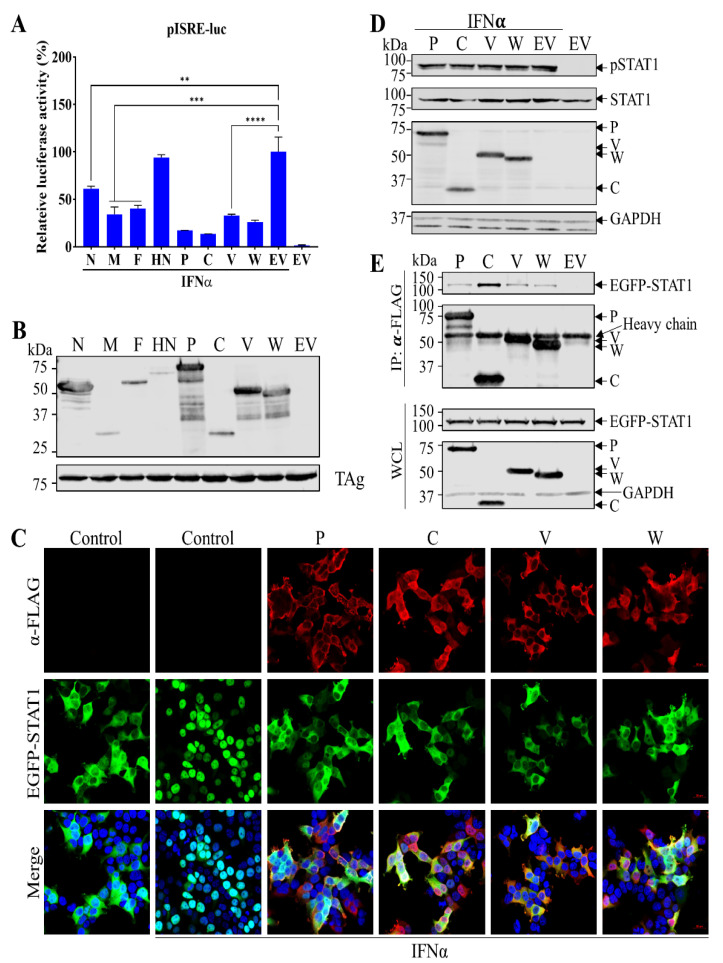
PRV1 P gene products inhibit the type I IFN signaling pathway by interaction with STAT1. (**A**) PRV1 proteins antagonize the type I IFN signaling pathway. HEK293T cells cultured in 24-well plates were co-transfected with a plasmid expressing PRV1 proteins, or the empty vector (EV), pRL-SV40, and a luciferase reporter plasmid pISRE-Luc. At 24 h post-transfection, cells were treated with 100 units/mL of human IFNα for 16 h to stimulate the production of ISGs. Firefly and Renilla luciferase activities were measured with cell lysate. Relative luciferase activity is the ratio of firefly luciferase reporter activity to Renilla luciferase activity. The relative luciferase activity of EV with stimulation was set as 100%. Each data point shown represents a mean value from two biological replicates, and each experiment was repeated at least three times. Error bars show standard deviations of the normalized data. The expression of PRV1 proteins in HEK-293T cells was evaluated by Western blot analysis with anti-FLAG mAb M2. The SV40 larger T antigen (TAg) was detected as the loading control (**B**). (**C**) The nucleus translocation of STAT1. HEK-293T cells were co-transfected with a plasmid expressing P gene products, or the empty vector (EV), and a plasmid expressing EGFP-STAT1. At 24 h post-transfection, cells were treated with 100 units/mL human IFN-α for 2 h or mock-treated. Cells only expressing EGFP-STAT1 and treated with human IFNα were used as the positive control, whereas cells only expressing EGFP-STAT1 and mock-treated were used as the negative control. The scale bar is 20 μm. (**D**) HEK-293T cells were transfected with a plasmid expressing individual P gene products or the empty vector (EV). At 24 h post-transfection, cells were treated with 100 units/mL of human IFNα for 2 h. Western blot analysis with whole-cell lysate (WCL) was performed to detect STAT1 and phosphorylated STAT1 (pSTAT1) using specific mAbs listed in MATERIALS AND METHODS. The expression of P gene products was also verified by Western blot analysis, and GAPDH was detected as the loading control. (**E**) HEK-293T cells were co-transfected with a plasmid expressing P gene products, or the empty vector (EV), and a plasmid expressing EGFP-STAT1. P gene products were immunoprecipitated with anti-FLAG mAb M2. Western blot analysis with IP eluents and whole-cell lysate (WCL) was conducted to detect EGFP-STAT1 and P gene products. GAPDH was detected as the loading control. **, *p*-value < 0.01; ***, *p*-value < 0.001; ****, *p*-value < 0.0001.

Since PRV1 infection is unable to induce RIG-I and ISG15 expression (Figure 1C,D), we hypothesized that PRV1 proteins could inhibit the JAK-STAT signaling pathway, which leads to hundreds of interferon-stimulated genes (ISGs). To identify PRV1 proteins that can inhibit the expression of ISGs, we used the pISRE-Luc reporter plasmid in which the firefly luciferase gene is under the control of the ISRE promoter. HEK-293T cells were co-transfected with a plasmid expressing individual PRV1 proteins (N, P, F, HN, P, C, V, and W), pISRE-luc reporter plasmid, and pRL-SV40. The ISRE promoter was stimulated with IFNα treatment. As expected, the absence of PRV1 protein expression activation of ISRE promoter was evident by enhanced relative firefly luciferase activity. HN protein showed no inhibitory effect on the ISRE promoter. In contrast, several PRV1 proteins (N, M, F, P, C, V, and W) significantly suppressed the activation of the ISRE promoter, indicated by a 40~90% reduction of relative luciferase activity (Figure 5A).

Since four PRV1 proteins (P, C, V, and W) encoded by the P gene own a strong suppression effect on the ISRE promotor, we further explored the mechanism. With the stimulation of IFNα, the STAT1 is phosphorylated, forms a complex with other factors, and translocates from the cell cytoplasm into the nucleus, where it binds to the ISRE promoter to activate the expression of ISG genes. Initially, the translocation of STAT1 was analyzed in cells co-transfected with a plasmid expressing STAT1-EGFP fusion protein and a plasmid expressing individual P gene products. At 24 hpt, cells were treated with IFNα for 2 h and fixed for immunofluorescence assay, which detects P gene expression. As expected, EGFP-STAT1 was mainly localized in cell cytoplasm without IFNα stimulation, and its nucleus translocation was stimulated in cells without viral protein expression by IFNα (Figure 5C). In contrast, in cells with P gene products expression, IFNα was unable to induce EGFP-STAT1 nucleus translocation, indicating that P gene products inhibit EGFP-STAT1 nucleus translocation or the upstream steps of the JAK-STAT signaling pathway.

To further characterize the mechanism, the phosphorylation status of STAT1 was determined in HEK-293T cells with the expression of individual P gene products. IFNα treatment for 2 h was performed to activate the JAK-STAT signaling pathway. Total STAT1 and phosphorylated STAT1 (pSTAT1) were detected by Western blot analysis with specific mAbs. Similar levels of total STAT1 were observed in all cell lysates, whereas pSTAT1 was only detected in cells with IFNα treatment (Figure 5D). No obvious difference in pSTAT1 expression level was observed among cells with the absence or the presence of P gene products expression, suggesting that STAT1 phosphorylation is not affected by PRV1 P gene products.

Next, since the colocalization between EGFP-STAT1 and P gene products was observed (Figure 5C), we hypothesized that STAT1 is sequestered in cytosol by interaction with P gene products. To test it, a coimmunoprecipitation (co-IP) assay was performed using cell lysates with coexpression of EGFP-STAT1 and individual P gene products. The cell lysate with only EGFP-STAT1 expression was used as a control. An anti-FLAG mAb was used to pulldown FLAG-tagged P gene products and their interacting partners. Western blot analysis was performed with the eluents to detect EGFP-STAT1 and P gene products with specific mAbs. The equal expression levels of EGFP-STAT1 were also verified. As expected, EGFP-STAT1 was not detected in the control sample (Figure 5E; lane “EV”). In contrast, EGFP-STAT1 was detected in all co-IP reactions with P gene expression (Figure 5E), indicating that P gene products can interact with STAT1. Therefore, our results suggested that P gene products inhibit the nucleus translocation of STAT1 via interacting with STAT1 but not via blocking STAT1 phosphorylation in the JAK-STAT signaling pathway.

## 4. Discussion

PRV1 was initially identified in nasopharyngeal samples of slaughterhouse pigs in Hong Kong [8] and is currently widespread in many countries on three continents [3,9,10,13,14,15,17]. To date, two PRV1 strains have been isolated from pig nasal swabs and characterized in vitro by two research groups in the USA [16,17]. The pathogenesis of the first PRV1 strain (USA/MN25890NS/2016) was evaluated in conventional and colostrum-deprived/cesarean-derived pigs [18]. This virus established infection in the upper and lower respiratory tracts and caused mild macroscopic lung lesions in conventional pigs but not in colostrum-deprived/cesarean-derived pigs. Mild microscopic lung lesions were observed in both conventional and colostrum deprived/cesarean-derived pigs. This pig challenge study suggested that PRV1 may not be a primary agent involved in the porcine respiratory disease complex. However, given PRV1’s genetic diversity and co-infection with other porcine respiratory agents [3,11,12], it is still unknown whether other genetically different strains could cause severe clinical outcomes and whether PRV1 could enhance porcine respiratory disease caused by other infectious agents. Paramyxoviruses utilize diverse strategies to evade the first defensive line driven by IFN in host cells [19]. Since PRV1 is a member of the *Respirovirus* genus in the *Paramyxoviridae* family, we hypothesized that PRV1 also suppresses host IFN production and signaling pathways to benefit its replication and the infection of secondary pathogens in porcine respiratory disease. In this study, we investigated the mechanisms of PRV1 in arresting host type I IFN production and signaling in viral-infected cells and ecotopic overexpression systems.

Paramyxoviruses are generally weak inducers of type I IFN, although virus stocks rich in defective interfering particles are used as IFN inducers [40,41]. As expected, PRV1 stimulated very limited production of type I IFN and ISGs and significantly suppressed their production induced by SeV infection. The P gene of the *Paramyxoviridae* family encodes different nonstructural proteins, such as C, V, W, D, and I, in addition to the P protein. These P gene products have been identified as IFN antagonists, and the V protein is the best characterized [19]. The highly conserved cysteine-rich zinc-binding domain at the C-terminus of the V protein selectively interacts with and antagonizes the pattern recognition receptors (MDA5 and RIG-I) and downstream factors in the type I IFN induction cascades [42]. In line with previous studies, all four P gene products of PRV1 suppressed the activation of the IFNβ promoter. The highly conserved cysteine-rich zinc-binding domain at the C-terminus of the V protein was also identified at the C-terminus of the PRV1 V protein. Not surprisingly, PRV1 V protein inhibited the IFNβ promoter activity induced by MDA5 and RIG-I but not by MAVS, suggesting that MDA5 and RIG-I are targeted by the V protein. MDA5 was first identified as a major target of the V proteins of several paramyxoviruses, and the cysteine-rich zinc-binding domains of the V proteins mediate the interaction [30,33,35]. We also observed the interaction between the PRV1 V protein and MDA5. Whether the C-terminal cysteine-rich domain mediates the interaction should be explored in the future. The V protein of the Nipah virus suppresses RIG-I activation by preventing the ubiquitination of RIG-I by TRIM25, which is mediated by the interactions between the V protein and RIG-I/TRIM25 [37]. This mechanism was also confirmed with V proteins of SeV, MeV, and HPIV5. Here, the ubiquitination of RIG-I was also inhibited by the PRV1 V protein, and the interaction between the PRV1 V protein and RIG-I/TRIM25 was confirmed. However, the critical region of the PRV1 V protein involved in this mechanism needs to be determined.

Besides antagonizing IFN production, paramyxovirus V proteins suppress the IFN signaling pathway by targeting the STATs proteins [43]. The phosphorylation and translocation to the nucleus of STAT1 and STAT2 could be prevented by the V proteins. For PRV1, all four P gene products own the antagonism effect on the IFN signaling pathway. Their interactions with STAT1 were detected, while the phosphorylation of STAT1 was not affected by these P gene products. Furthermore, the nucleus translocation of STAT1 was blocked by these P gene products. Since several P gene products are not essential for the viability of paramyxoviruses, we will further confirm their antagonism function on IFN production and signaling pathway using our PRV1 reverse genetics [17] to construct recombinant viruses with these products knockout individually.

Based on luciferase reporter assays, PRV1 M and N proteins exhibited antagonism function on type I IFN production and signaling pathway. As a structural protein, the M protein mainly drives viral assembly and budding. Recently, the antagonism effect on type I IFN production and signaling pathway was demonstrated for the M proteins of the Nipah virus and HPIV3 [44,45]. The N protein encoded by the N gene forms a coiled nucleocapsid structure around the viral RNA called ribonucleoprotein complex (RNP), which associates with the viral RNA-dependent RNA polymerase complexes constituted by the P phosphoprotein and L polymerase protein. Since P and N proteins closely interact during viral replication, the N protein’s function as an IFN antagonist was explored for several paramyxoviruses [46,47]. Thus, screening IFN antagonists of PRV1 in this study generated similar results to previous studies on paramyxoviruses. The mechanisms employed by the PRV1 M and N to antagonize IFN responses should be further investigated.

## 5. Conclusions

PRV1 exhibited strong inhibitory effects on the production of IFNβ and ISGs, which may be related to its pathogenicity in the porcine respiratory disease complex. We identified several PRV1 proteins as antagonists to type I IFN production and signaling, including M, N, P, V, C, and W. The P gene products disrupted both IRF3 and NF-κB dependent type I IFN production and blocked type I IFN signaling by sequestering STAT1 in the cytoplasm. V protein blocked RIG-I polyubiquitination through interaction with TRIM25 and RIG-I and also bound to MDA5. These findings indicate that PRV1 antagonizes host innate immune responses using various mechanisms, which provides important insights into the pathogenicity of PRV1.

## Figures and Tables

**Table 1:** The oligonucleotides used in this study.

Name	Sequence (5′ to 3′)	Usage
3FLAG-HindIII-N-F	aaAAGCTTATGGCAGGGTTATTAAGTGT	The expression plasmid for N
BamHI-N-R	aaGGATCCTTATATTCCTCCTAGTGCATTCAT	
3FLAG-HindIII-P-F	aaAAGCTTATGGATCAGGAcGCCCTC	The expression plasmid for P
SalI-P-R	ggGTCGACTTATTCATTACTTGATTCTATATCTTCCTC	
3FLAG-HindIII-C-F	ggAAGCTTATGCCCTCTTTTCTGAAGAA	The expression plasmid for C
SalI-C-R	ttGTCGACCTACTCTTGGATTATGTGTGC	
PRV1-F-SacI-F	aaGAGCTCgccaccATGCAAATCATCATCCTCAGACC	The expression plasmid for F
PRV1-F-FLAG-R	aaCTCGAGCTACTTGTCGTCATCGTCTTTGTAGTCTCCCATGAAATTAGTAGGC	
PRV1-M-SacI-F	aaGAGCTCgccaccATGGCCGAGATCTACAAGT	The expression plasmid for M
PRV1-M-FLAG-R	aCTCGAGCTACTTGTCGTCATCGTCTTTGTAGTCAACTTTTATTTTCCCAATATTTTTTG	
PRV1-HN-SacI-F	aaGAGCTCgccaccATGGAAGAGACCAAAGTTAAG	The expression plasmid for HN
PRV1-HN-FLAG-R	aaCTCGAGTTACTTGTCGTCATCGTCTTTGTAGTCTAAATTGCTTATCCTGCAA	
V-F	GATTGGTAAAAAGGGGCACAGAAGAGAATAC	The expression plasmid for V
V-R	GTATTCTCTTCTGTGCCCCTTTTTACCAATC	
W-F	GATTGGTAAAAAGGGGGCACAGAAGAGAATAC	The expression plasmid for W
W-R	GTATTCTCTTCTGTGCCCCCTTTTTACCAATC	

## Data Availability

The datasets generated for this study are available to the corresponding author upon request.
